# Mothers of Autistic Children: Lower Plasma Levels of Oxytocin and Arg-Vasopressin and a Higher Level of Testosterone

**DOI:** 10.1371/journal.pone.0074849

**Published:** 2013-09-25

**Authors:** Xin-Jie Xu, Xiao-Jing Shou, Jin Li, Mei-Xiang Jia, Ji-Shui Zhang, Yan Guo, Qing-Yun Wei, Xiu-Ting Zhang, Song-Ping Han, Rong Zhang, Ji-Sheng Han

**Affiliations:** 1 Neuroscience Research Institute & Department of Neurobiology, School of Basic Medical Sciences, Peking University, Beijing, China; 2 Key Laboratory for Neuroscience, Ministry of Education/National Health and Family Planning Commission, Peking University, Beijing, China; 3 Mental Health Institute, Peking University, Beijing, China; 4 Department of Neurology and Center of Rehabilitation, Beijing Children’s Hospital, Capital University of Medical Sciences, Beijing, China; 5 Department of ophthalmology, Beijing University of Chinese Medicine Third Affiliated Hospital, Beijing, China; 6 Beijing Yangguang Youyi Rehabilitation Center, Beijing, China; 7 Beijing Tongkang Rehabilitation Center, Beijing, China; 8 HANS International Incorporated, Belle Mead, New Jersey, United States of America; Rutgers University, United States of America

## Abstract

**Background:**

Autism is a pervasive neurodevelopmental disorder,thought to be caused by a combination of genetic heritability and environmental risk factors. Some autistic-like traits have been reported in mothers of autistic children. We hypothesized that dysregulation of oxytocin (OXT), Arg-vasopressin (AVP) and sex hormones, found in autistic children, may also exist in their mothers.

**Methods:**

We determined plasma levels of OXT (40 in autism vs. 26 in control group), AVP (40 vs. 17) and sex hormones (61 vs. 47) in mothers of autistic and normal children by enzyme immunoassay and radioimmunoassay, respectively and investigated their relationships with the children’s autistic behavior scores (Childhood Autism Rating Scale (CARS) and Autism Behavior Checklist (ABC)).

**Results:**

Significantly lower plasma concentrations of OXT (*p*<0.001) and AVP (*p*<0.001), as well as a higher level of plasma testosterone (*p*<0.05), were found in mothers of autistic children vs. those of control. The children’s autistic behavior scores were negatively associated with maternal plasma levels of OXT and AVP.

**Conclusions:**

These results suggest that dysregulation of OXT, AVP and/or testosterone systems exist in mothers of autistic children, which may impact children’s susceptibility to autism.

## Introduction

Autism is a pervasive neurodevelopmental disorder characterized by various degrees of impairment in language, communication and social skills, and repetitive and stereotypic patterns of behavior [1]. The exact etiology of autism is unknown and no specific biomarker for autism has been found yet [2], [3]. So far autism is still regarded as a disorder spectrum caused by a combination of genetic heritability and environmental risk factors [4], [5]. The intrauterine environment and biochemical interactions between mother and fetus may impact susceptibility of the children to autism.

The contribution of oxytocin (OXT) and Arg-vasopressin (AVP) to mammalian social behavior and their potential roles in autism has been studied extensively over the past decade (for reviews see [6], [7]). Accumulated evidence suggests that dysregulation of these two neuropeptide systems may be involved in the development of autism [6], [8], [9], [10], [11], [12], [13]. Lower plasma levels of OXT and AVP were found to be associated with autistic traits [9], [10], and intranasal or intravenous administration of OXT could improve social cognition and behaviors not only in autistic patients [14], [15], [16] but also in healthy volunteers [17], [18]. Several studies have reported that mothers of autistic children had more autistic-like traits compared to mothers of normal children [19], [20], [21]. Therefore, it is hypothesized that dysregulation of OXT and AVP systems may exist in mothers of autistic children.

Molecular and behavioral evidence also points to a connection between dysregulation of sex-steroid hormones and the onset or development of autism [22], [23], [24], [25]. Increased levels of blood androgen have been found in autistic children [25] and adults [22]. Furthermore, fetal exposure to elevated levels of androgen may increase susceptibility towards autism [26], [27]. Children born from hyperandrogenic women (such as polycystic ovary syndrome (PCOS) or severe acne) have been reported to have more autistic traits and are at higher risk for autism [28], [29]. Thus, it is hypothesized that sex hormone abnormalities may exist in mothers of autistic children.

The objective of the present study was to examine whether there are changes in maternal plasma levels of OXT, AVP, testosterone or estradiol, and to investigate the relationships between the biochemical statuses of the mothers and children’s autistic behavior scores. The findings in this study may provide useful information for the understanding of autism.

## Materials and Methods

### Ethics Statement

The study was approved by the Peking University Institutional Review Board (IRB00001052-11080). Detailed information on the aims and protocols of the study were explained to all mothers involved in the study. All mothers signed the written form of full informed consent in order to participate.

### Participants

Autistic children and their mothers were recruited from two autism rehabilitation centers (Wucailu Rehabilitation Center and Yangguang Youyi Rehabilitation Center for Children with Autism, Beijing, China). The diagnosis of autism was made by an experienced psychiatrist (MXJ) in children with autism spectrum disorders using the Diagnostic and Statistical Manual of Mental Disorders-IV-Text Revision (DSM-IV-TR, 2000). Autistic children with symptoms of other psychiatric disorders were excluded. Children in the control group and their mothers were recruited through advertisements. The enrollment screen was conducted by an experienced psychiatrist (JSZ) through parent interview. Children were excluded from the control group if they have psychiatric conditions or other potentially confounding medical conditions. All the children enrolled were between 2 to10 years old.

Inclusion criteria for mothers in both groups included: 1) normal functioning in daily life; 2) no past or present history of any psychiatric disorder; 3) no significant medical illness; 4) before menopause with menstrual cycle; 5) not being pregnant or lactating or using oral contraceptives.

### Assessment of Autistic Symptom

The following two scales were used to assess autistic symptom in autistic and normal children:

#### 1. Childhood Autism Rating Scale (CARS)

A 15-item behavior rating scale consists of 14 domains that are generally affected by severe autism, plus one category of general impressions of autism [30]. It is widely used by psychiatrists during diagnosis of autism.

#### 2. Autism Behavior Checklist (ABC)

A behavior checklist consists of 57 items in 5 categories: sensory, relating, body and object use, language, and social and self-help [30]. The scale utilizes an observer’s rating of the child’s behavior to quantify behaviors typically associated with autism.

### Blood Sample Collection

Blood samples were collected by trained nurses between 8∶00 and 10∶30 a.m. Mothers were previously informed to fast overnight and allowed only moderate amount of drinking water to minimize the potential effects of food and water intake. The phase of the menstrual cycle was recorded. Ten milliliters of venous blood was collected into chilled EDTA tubes containing aprotinin (500 KIU/mL blood). The samples were then centrifuged at 1,600 g for 15 minutes at 4°C. Plasma was isolated and divided into 700 µL aliquots and immediately frozen at −80°C until assay.

### Biochemical Analyses

#### 1. Measurements of OXT and AVP concentrations

Samples for OXT and AVP concentration measurements were collected between June 2012 and October 2012. Plasma OXT and AVP levels were determined by enzyme immunoassay (EIA) (Enzo Life Sciences, PA, USA) after prior extraction with acetone and petroleum ether [9]. The assays were performed according to the manufacturer’s instructions. The EIAs were highly sensitive (limit of detection: 11.7 pg/mL for OXT and 3.39 pg/mL for AVP) with very little cross-reactivity with other related compounds. The cross-reactivity between AVP and OXT was less than 0.2%. The r-values of standard curves were greater than 0.999 for both assays.

#### 2. Measurements of testosterone and estradiol concentrations

Samples for testosterone and estradiol measurements were collected between September 2011 and August 2012. Testosterone and estradiol levels were determined by radioimmunoassay (RIA) (North Institute of Biological Technology, Beijing, China). The sensitivities of assay for testosterone and estradiol were 20 pg/mL and 5 pg/mL, respectively, with very little cross-reactivity with other steroid hormones. The r-values of standard curves were greater than 0.99 for both assays.

Analyses were conducted blindly in respect to which group the samples belonged to. For each assay, all of the samples were run at the same time.

### Statistics

Statistical analyses were performed with Statistical Package for the Social Science version 19.0 (SPSS Inc., Chicago, Illinois) and GraphPad Prism version 5.0 (GraphPad Software Inc., San Diego, CA). The initial analysis examined the participant demographics of the two groups with the unpaired *t* test (age, menstrual cycle) and Mann–Whitney *U* test (CARS, ABC). Continuous data were checked for normal distribution using the Shapiro-Wilk test first. For those data that were not normally distributed, non-parametric tests (Mann–Whitney U test) were used for unpaired comparison between groups. Linear regression analysis was used to examine the correlation between the log-transformed plasma levels of OXT and AVP. The Spearman correlation coefficient (*Rho*) was calculated to assess the association between maternal biochemical levels and children’s behavior scores. The differences in occurrence of risk factors during pregnancy and birth between groups were analyzed using the χ^2^ test. For all tests, a value of *p*<0.05 (two-tailed) was considered statistically significant.

## Results

### Group Characteristics

A total of 109 pairs of mothers and children (62 in autism, 47 in control group) met the inclusion criteria and were enrolled in this study. Descriptive statistics for baseline characteristics are provided in Table 1. The two groups were well matched for maternal chronological age, pregnant age and menstrual cycle days. As expected, autistic children showed significantly higher scores in CARS and ABC than normal children.

**Table 1:** Participant Demographics.

	Autism	Control
n	62	47
Mothers		
Age, y	31.02(4.83)	30.54(3.03)
Pregnant age, y	26.71(4.47)	25.79(3.31)
Menstrual cycle, d	29.31(3.81)	29.81(3.03)
Children		
Age, y	4.51(1.71)	4.69(1.19)
CARS^a^	36.51(4.81)	15.64(0.80)
ABC		
Sensory^a^	8.55(5.01)	0.91(1.86)
Relating^a^	15.28(8.42)	0.97(2.07)
Body and object use^a^	8.38(6.10)	2.88(4.34)
Language^a^	14.05(7.34)	1.53(2.50)
Social and self-help^a^	11.95(4.86)	2.94(3.33)
Total^a^	58.20(23.14)	9.22(10.53)

Abbreviations: CARS, Childhood Autism Rating Scale; ABC, Autism Behavior Checklist.

Data are presented as mean (standard deviation, SD) unless otherwise indicated.

a *p*<0.001.

Blood samples for sex hormone analysis were collected from 108 mothers (61 in autism, 47 in control group), of which 86 samples (40 in autism, 26 in control group) were tested for OXT and AVP concentrations. For mothers who took part in blood sample analysis, no significant difference was found in the baseline characteristics between the two groups of autism and control, respectively (data not shown).

### Plasma OXT and AVP Levels

**Figure 1:**
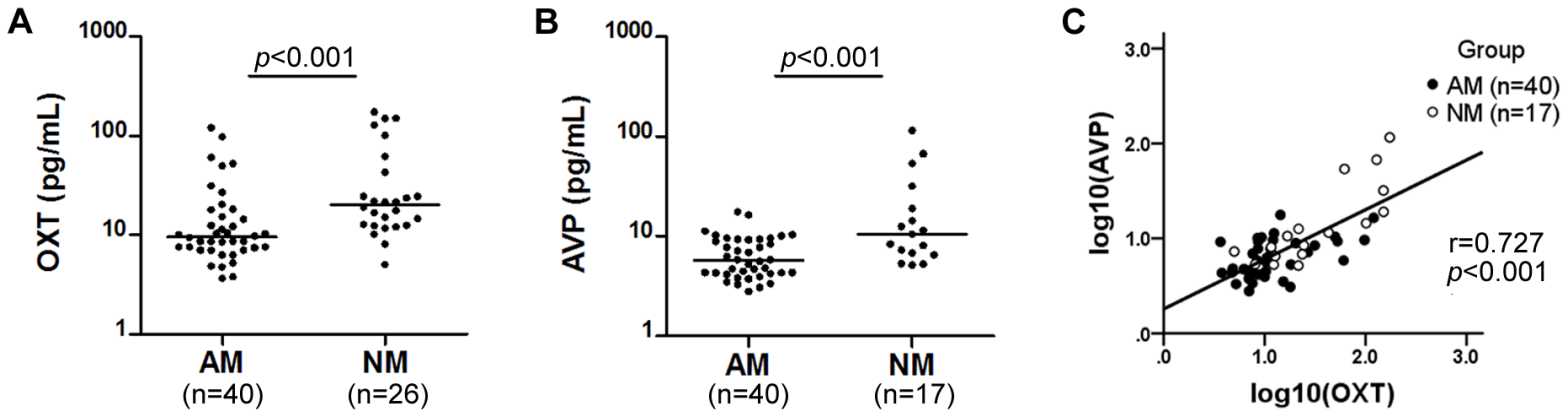
Plasma OXT and AVP levels and linear regression analysis. Median value for each group is shown by a horizontal bar. Mothers of autistic children displayed a significantly lower plasma level of OXT when compared to mothers of normal children (**A**). Plasma AVP levels were also significantly reduced in mothers of autistic children compared to mothers of normal children (**B**). A positive correlation was shown between log-transformed plasma OXT levels and log-transformed plasma AVP levels (r = 0.727, *p*<0.001) (**C**). AM: mothers of autistic children; NM: mothers of normal children; OXT: oxytocin; AVP: Arg-vasopressin.

Pronounced differences in plasma OXT and AVP levels were found between mothers of autistic children (AM) and mothers of normal children (NM). AM demonstrated a significantly lower level of plasma OXT (9.56 pg/mL, interquartile range (IQR) 7.12–17.29) compared with NM (20.22 pg/mL, IQR 12.43–47.63, *p*<0.001) (Fig. 1A). There was also a significantly reduced level of plasma AVP in AM (5.76 pg/mL, IQR 4.25–9.30) in comparison with NM (10.58 pg/mL, IQR 6.66–25.49, *p*<0.001) (Fig. 1B). Furthermore, plasma levels of OXT and AVP showed a significant positive correlation with each other (r = 0.727, *p*<0.001) (Fig. 1C). Blood samples collected from 9 mothers who reported to have had breakfast before the blood collection were excluded from the AVP analysis since food and water intake are known to impact plasma AVP level [31].

### Plasma Testosterone and Estradiol Levels

**Figure 2:**
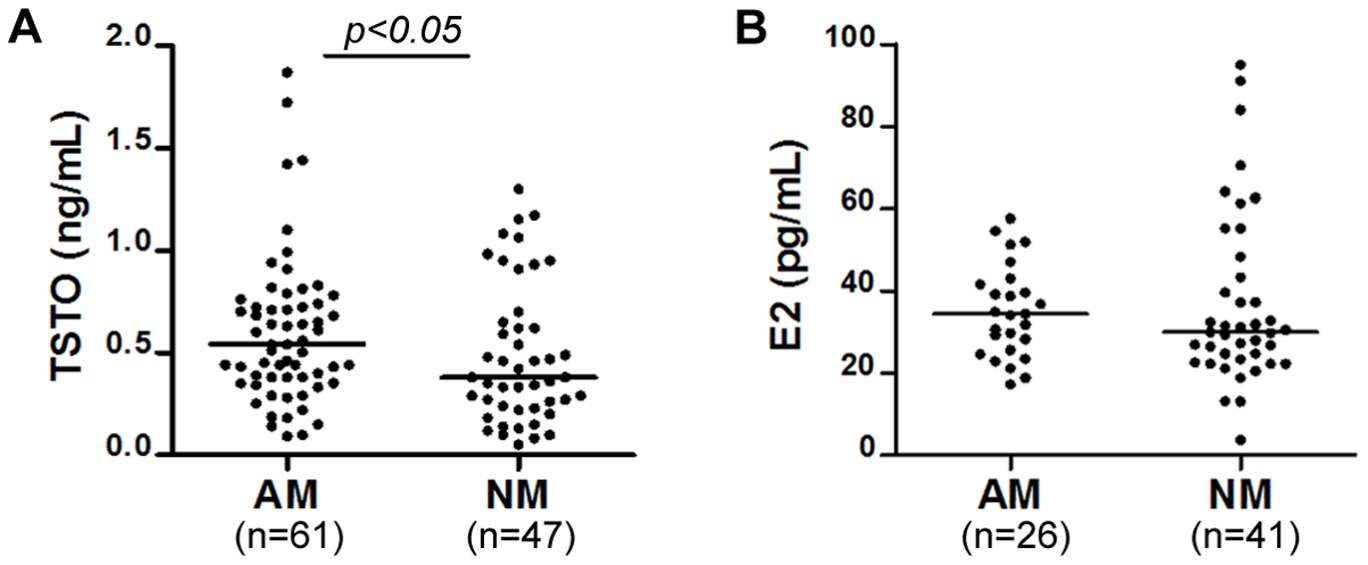
Plasma testosterone and estradiol levels. Median value for each group is shown by a horizontal bar. Elevated plasma testosterone levels were found in mothers of autistic children as compared to mothers of normal children (**A**). Plasma estradiol levels showed no difference between the two groups (**B**). AM: mothers of autistic children; NM: mothers of normal children; TSTO: testosterone; E2: estradiol.

Statistically significant elevation in plasma testosterone levels was found in AM (0.540 ng/mL, IQR 0.365–0.730) compared with NM (0.380 ng/mL, IQR 0.230–0.650, *p*<0.05) (Fig. 2A). Only mothers who were in follicular phase (within 7 days from the beginning of menstruation) were included for estradiol analysis since plasma estradiol level fluctuates naturally during the menstrual cycle. There was no statistically significant difference in plasma estradiol level between the two groups (AM: 34.28 pg/mL, IQR 25.34–41.91; NM: 29.96 pg/mL, IQR 22.96–45.81, *p*>0.05) (Fig. 2B). No significant correlation was found between plasma testosterone levels and estradiol levels.

### Relationships between Maternal Hormonal Levels and Children’s Behavior Scores

The Spearman correlation analysis was used to assess the relationships between maternal hormonal levels and children’s behavior scores. The correlation coefficient (*Rho*), significance (*p*) and valid number of samples (N) are shown in Table 2.

**Table 2:** Correlation analysis between maternal biochemical levels and children’s behavior scores.

		CARS	ABC					
			Sensory	Relating	Body and object use	Language	Social andself-help	Total score
Oxytocin	*Rho*	**−.399**	**−.305**	**−.421**	**−.374**	**−.421**	**−.360**	**−.426**
	*p*	**.001**	**.021**	**.001**	**.004**	**.001**	**.006**	**.001**
	n	**61**	**57**	**57**	**57**	**57**	**57**	**57**
Arg-Vasopressin	*Rho*	**−.314**	**−**.199	**−.418**	**−**.281	**−.448**	**−.305**	**−.365**
	*p*	**.023**	.176	**.003**	.053	**.001**	**.035**	**.011**
	n	**52**	48	**48**	48	**48**	**48**	**48**
Testosterone	*Rho*	.062	**−**.037	.011	**−**.092	−.120	−.004	−.069
	*p*	.573	.760	.926	.443	.318	.971	.566
	n	85	71	71	71	71	71	71
Estradiol	*Rho*	−.057	.021	.009	−.005	−.073	−.054	.011
	*p*	.668	.885	.951	.974	.607	.702	.938
	n	58	52	52	52	52	52	52

Abbreviations: CARS, Childhood Autism Rating Scale; ABC, Autism Behavior Checklist.

**Figure 3:**
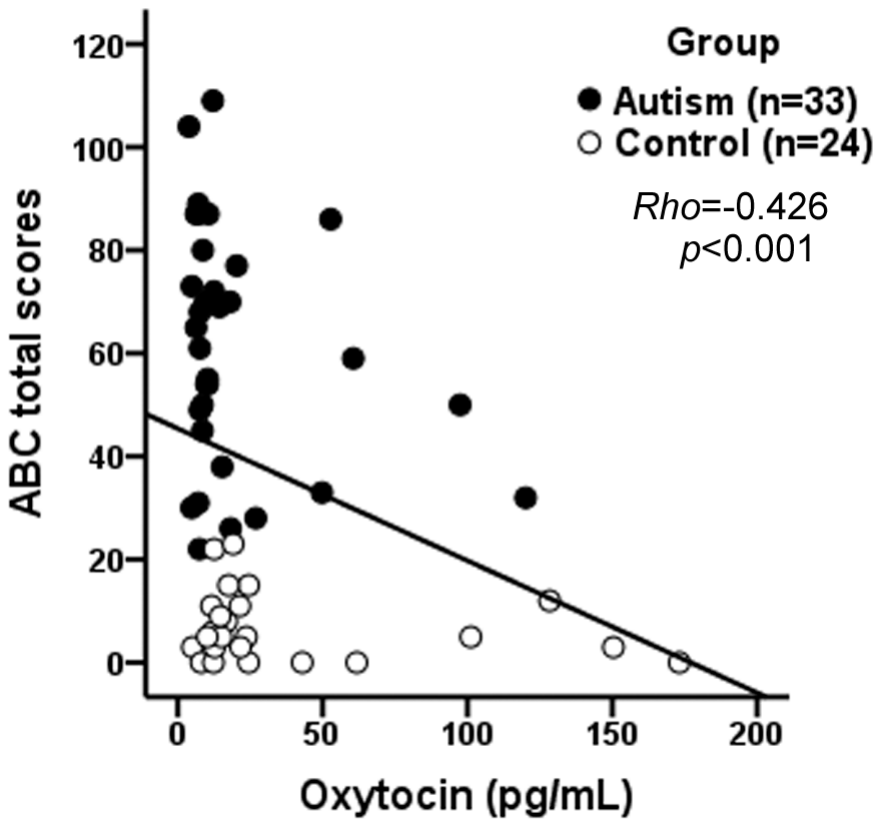
Scatter-plot between maternal plasma OXT levels and children’s ABC total scores. Increasing maternal plasma OXT levels is associated with decrease in children’s ABC total scores (Spearman rank order correlation, *Rho = −0.426, p*<0.001). OXT: oxytocin; ABC: Autism Behavior Checklist.

Maternal plasma OXT levels showed significant negative correlations with children’s CARS and ABC subscale (sensory, relating, body and object use, language, and social and self-help) and total scores. As a representative, the ABC total scores of all the children were plotted against their mothers’ OXT levels and depicted in Fig. 3. Significant negative correlations were also found between maternal plasma AVP levels and children’s CARS and ABC subscale (relating, language, and social and self-help) and total scores.

In contrast to the neuropeptides, there were no significant correlations between maternal plasma testosterone or estradiol levels and children’s behavior scores.

## Discussion

Autism has received more attention around the world in recent years due to its relatively high prevalence, poor prognosis and absence of effective therapies [1]. Recent studies suggest that autism should be considered as highly heterogeneous complex disorders (from etiology to symptom) and it is caused by a combination of genetic heritability and environmental risk factors [4]. Since the exact etiology of autism is not well understood, there is no effective prevention strategies available today.

In this cohort study, we have demonstrated significantly lower levels of OXT and AVP in AM as compared to NM. Additionally, maternal plasma OXT and AVP values were found to be significantly correlated with children’s autistic behavior scores. Concentration of testosterone levels was found to be elevated in mothers of autistic children, but showed no correlation with the severity of the children’s autistic syndrome. OXT and AVP are two important neuropeptides which play key roles throughout mammalian evolution in modulating complex social cognition and behaviors [6], [7], [8]. Previous studies have found that autistic children had lower plasma levels of OXT [8] and AVP [10] as compared to healthy peers. To our knowledge, this is the first study demonstrating that significantly lower plasma levels of OXT and AVP also existed in mothers of autistic children. Moreover, the correlation analysis among all mothers recruited in this study revealed that maternal plasma levels of OXT and AVP showed negative correlations with their children’s behavior scores. There seems to be some common causation shared between autistic children and their mothers that lead to the dysregulation of OXT and AVP in both of them. However, since the biochemical evaluation was carried out several years after pregnancy, we don’t know for sure whether the alterations in maternal OXT and AVP are causative of the children’s autistic behavior. In our preliminary study, plasma AVP levels were also found to be decreased in mothers of autistic children. The results were reported at a local neuroscience meeting [32].

Although the mean levels of maternal plasma OXT and AVP concentrations were significantly decreased in autistic group, the respective distributions of the two neuropeptides in the two groups were obviously overlapped. The overlapping distributions of the two neuropeptides, as revealed by other studies in autistic children [8], [10], remind us that multiple factors contribute to the development of autism and a simple deficit of OXT or AVP is far from enough to interpret the etiology of this highly heterogeneous illness. In addition, changes in plasma OXT and AVP primarily reflect changes in the synthesis and release of these neuropeptides. Abnormalities in other aspects of the OXT and AVP systems, such as the receptor binding and intracellular transduction mechanism as well as the downstream transmitters and modulator systems should also be considered.

OXT and AVP are “twin” nonapeptides with only 2 amino acids different between them [6]. The two neuropeptides are thought to arise from a gene-duplication event through evolution and most invertebrates have only one oxytocin/vasopressin homolog, whereas vertebrates have two [33]. In mammals, they are both synthesized in the hypothalamus supraoptic and paraventricular nuclei and stored in the posterior pituitary for release [6]. One of the interesting findings in our study was that levels of OXT and AVP in maternal plasma showed a significant positive correlation with each other (Fig. 1C), as if they were released from the same neurons, although there were enough evidence showing that these two neuropeptides were synthesized and released by different kinds of neurons [6], and being regulated by different types of mechanisms [6], [31]. Though it remains to be established, it seemed that in the basal physiological status, the synthesis, release and metabolism of these two neuropeptides may in some way be related.

One of the major concerns is whether the concentration of peripheral OXT and AVP levels parallel that in the central nervous system. Although a strict parallelism remains to be established, several lines of evidence suggest that plasma level of OXT did at least partially reflect its central functioning [8], [34], [35], [36]. While magnocellular neurons in the supraoptic and paraventricular nuclei were known to send axons to the posterior pituitary for release of the neuropeptides into the blood, and parvocellular neurons were more or less paracrine in nature to release nonapeptides to the vicinity of the brain, these two neuronal components may work side by side. Compared to the OXT, the situation for AVP may be a little more complicated since the peripheral level of AVP (also known as the antidiuretic hormone) is greatly and quickly affected by intravascular volume and plasma osmolarity [31]. Therefore, it is critical to minimize the impact of food and water intake before sample collection. In the present study, mothers were previously informed to fast overnight and to have only moderate amount of drinking water before sample collection. Unfortunately, nine of them reported to have had breakfast before the blood collection. Their blood samples were therefore measured but excluded from the analysis. As can be expected, their plasma AVP levels were extremely low (Median 4.81 pg/mL, IQR 3.14–5.91, n = 9), compared to the rest of the mothers of normal children (Median 10.58 pg/mL, IQR 6.66–25.49, n = 17). Results obtained from these incidental cases confirmed that the plasma AVP levels are tightly associated with food and water intake. It reminded us that the instruction of “only moderate amount of drinking water is allowed” may also affect the level of AVP, at least in a moderate degree.

Sex hormones are very upstream regulators that have numerous functional targets. They play important and diverse roles in the regulation of structure and function of the central nervous system [37]. Prenatal androgen exposure can have long-standing and permanent effects on the structure and function of the fetal central nervous system [37]. Moreover, sex steroids seem to be part of the mechanisms regulating the brain’s oxytocinergic and vasopressinergic systems [6]. Lower levels of OXT and higher levels of testosterone has been associated with more autistic-like traits in healthy volunteers [17], [18], [28]. In the present study, mothers of autistic children showed higher levels of testosterone, but no changes were found in estradiol levels. Our results agree with previous studies in which higher risk for autism has been reported among children born from women with androgen-related disorders [28], [29], and provide additional evidence that maternal hyperandrogenism may be a risk factor for the onset and development of autism in children. This notion is supported by results of other studies in which fetal exposure to elevated levels of amniotic androgen may increase children’s susceptibility towards autism [26], [27]. Using PCOS patients as an elective biological model, Palomba *et al* demonstrated that children born from hyperandrogenic women seem to have a higher risk for PDDs, due probably to an unbalanced prenatal exposure to high levels of testosterone [29]. It is obvious that the amniotic androgen level is not sorely determined by the maternal androgen level, but also by the androgen produced by the fetus itself. The hypothesis that maternal testosterone levels may adjust testicular androgen production prenatally in the male fetus minimizing the androgen elevation, as suggested by Palomba *et al* [29], may also be a reason that no significant correlations can be found between maternal plasma testosterone levels and their children’s autistic behavior scores.

As a preliminary study there are several limitations ought to be mentioned. 1) The blood samples tested in this study were collected from the mothers when their children were already 3–5 years old. Results obtained in this study may not be representing the situation as those during their pregnancy. Therefore, further studies should be performed to observe the biochemical characteristics in mothers during their pregnancy, which will be more convincing to reflect the fetal developmental environment. 2) As an exploratory study, sex hormones were measured in plasma samples without calculating the total and free testosterone/estradiol levels respectively, which would obviously be more comprehensive and accurate. 3) Most of the participants (>90%) recruited in this study are Chinese Han population. The geographical distribution of autistic children are more scattered (coming from broad north China areas) as compared to that of the normal children, since the later were recruited mainly from kindergartens of the suburb of Beijing. These limitations should be considered when data are interpreted.

Despite these limitations and the preliminary nature of this study, our results suggest that dysregulation of OXT, AVP and testosterone in the mothers should be considered as candidate elements for the understanding and the prevention of the birth of autistic children.
